# Single-Chamber Cardiac Pacemaker Implantation in a Donkey with Complete AV Block: A Long-Term Follow-Up

**DOI:** 10.3390/ani11030746

**Published:** 2021-03-09

**Authors:** Markéta Sedlinská, Radovan Kabeš, Miroslav Novák, Filip Kološ, Pavlína Melková

**Affiliations:** 1 Equine Clinic, Faculty of Veterinary Medicine, University of Veterinary and Pharmaceutical Sciences Brno, Palackého tř. 1946/1, 612 42 Brno, Czech Republic; sedlinskam@vfu.cz (M.S.); kabesr@vfu.cz (R.K.); kolosf@vfu.cz (F.K.); 2 St. Anne’s University Hospital, Pekařská 53, 659 91 Brno, Czech Republic; 2608@mail.muni.cz

**Keywords:** arrhythmia, cardiac pacing, pacemaker pocket infection, donkey

## Abstract

**Simple Summary:**

Cardiac pacing is widely used in human and small-animal medicine for the treatment of many symptomatic bradyarrhythmias. Although the implantation of a pacemaker has previously been described in a horse and donkey, few data are available about the long-term follow-up. This article describes a pacemaker implantation in an African donkey with a complete AV block, and reports complications associated with this procedure, which included lead dislodgement and pacemaker pocket infection. After implantation, the donkey showed an improved quality of life and was used for the animal-assisted therapy of disabled children. The function of the pacemaker was checked regularly and, to date, the pulse generator has been replaced twice. Cardiac examination eighteen years after pacemaker implantation revealed that the pacemaker is working appropriately and no morphological changes were observed in the heart.

**Abstract:**

A five-month-old African jenny was presented with a history of exercise intolerance and syncopal episodes. Severe bradycardic arrhythmia due to a high-grade second-degree atrioventricular (AV) block with progression to complete AV block was diagnosed. The jenny underwent a transvenous single-chamber pacemaker implantation. The implantation procedure was performed in a lateral recumbency and the ventricular lead was inserted through the jugular vein. Positioning of the lead was guided by echocardiography. The pacemaker was programmed to VVI mode with a minimal ventricular rate of 40 pulses per minute, a pulse amplitude of 2.4 V, a pulse width of 0.5 ms and sensing amplitude of 2.5 mV. Short-term complications associated with the procedure included lead dislodgement and pacemaker pocket infection. The long-term outcome was satisfactory; the jenny showed improvement in heart function and quality of life after pacemaker implantation. The pulse generator replacement was performed twice (at nine-year intervals) and the intervention was always associated with a local inflammatory reaction around the pacing device. Cardiac examination 18 years after pacemaker implantation revealed no morphological changes in the heart; the electrode lead was still in the correct position and successful pacing and sensing of the ventricle were obtained. Regular follow-up checks are important to evaluate pacemaker function.

## 1. Introduction

Cardiac pacing is an effective therapy for various arrhythmias in humans and a therapeutic implantation of a cardiac pacemaker has also been described in a horse [1,2,3,4] and a donkey [5,6]. Indications for pacemaker implantation are symptomatic bradycardic arrhythmias such as third-degree AV block, high-grade second-degree AV block, sick sinus syndrome or persistent atrial standstill [1,3,5,6,7,8,9,10]. Transvenous pacemaker implantation is a relatively safe and simple procedure. Complications resulting from permanent pacemaker implantation are well-known in human and small-animal medicine and include lead malposition or displacement, pneumothorax, haemothorax, myocardial perforation, infection of pacemaker pocket, bacterial endocarditis and venous thrombosis [7,11,12,13]. In equids, pacemakers are not commonly used and little information is known about the long-term outcome. Hamir and Reef [14] described the complications and postmortal findings associated with a permanent transvenous pacing device in a horse thirty-four months after implantation. This article reports a pacemaker implantation in a five-month-old jenny, the short-term complications after intervention and an eighteen-year follow-up.

## 2. Materials and Methods

### 2.1. History and Clinical Findings

A five-month-old African jenny was presented to the Equine Clinic of the Veterinary University Brno with a history of exercise intolerance and frequent syncopal episodes. The syncopal episodes had started one month before and their frequency gradually increased. At the time of admission, short syncopal events (5–10 s) were observed approximately ten times per day.

The jenny was in good body condition (80 kg), bright and alert. Heart rate varied between 30 and 60 beats per minutes (bpm) and heart rhythm was irregularly irregular. No heart murmur was heard on auscultation. In both nostrils, a small amount of serous discharge was noticed. Respiratory rate was 24 breaths per minutes and lung sounds were normal. Neurological examination revealed no abnormalities. The complete blood count and biochemistry were unremarkable.

### 2.2. Electrocardiography and Cardiac Ultrasound 

**Figure 1:**
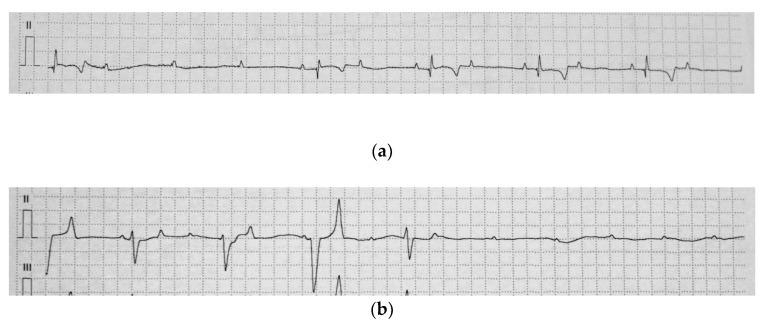
Base apex electrocardiogram of the jenny (**a**) A high-grade, second-degree atrioventricular (AV) block. Three consecutive P-waves are not followed by a QRS complex (**b**) A complete AV block with an independent atrial and ventricular rate. The tracing shows polymorphic QRS complexes and the interruption of the ventricular escape rhythm (25 mm/s, 1 cm/mV).

An electrocardiogram (ECG) showed a resting heart rate of 24 bpm with a high-grade second-degree AV block (Figure 1a). Impulse transmission from atria to ventricles was variable, with a conduction ratio of up to 13:1. The heart rate increased with physical activity or during manipulation with the animal. If the sinus rate increased over 80 bpm, a third-degree AV block occurred and complete atrial and ventricular dissociation was present. A junctional rhythm of a frequency around 50 bpm was associated with polymorphic ventricular complexes (Figure 1b). When the frequency of the ventricles was too low (pauses longer than 10 s), the syncope had occurred. 

A transthoracic echocardiogram was performed using a 1.5–3.6 MHz transducer (Eub-6500, Hitachi, Tokyo, Japan). Two-dimensional and M-mode echocardiography did not reveal gross morphological or functional abnormalities. No valvular regurgitation was found; echogenicity of the endocardium and myocardium was normal. On the basis of these findings, a diagnosis of atrioventricular block of unknown aetiology was established.

## 3. Treatment

### 3.1. Medical Treatment

The jenny was treated with phenylbutazone (2.2 mg/kg p.o. q12 h) and box-rested for 14 days. The incidence of syncopes during the treatment gradually decreased until they disappeared completely. However, on the ECG, the third-degree AV block was still present. The medication was discontinued but, on the fourth day after this, the syncopes reappeared. In view of this fact, the need for a pacemaker implantation was indicated.

### 3.2. Surgery

The jenny was premedicated with penicillin (30,000 IU/kg i.v.) and gentamicin (6.6 mg/kg i.v.), along with atropine (0.05 mg/kg i.m.), and placed under general anesthesia in a lateral recumbency position. Anesthesia was induced with diazepam (0.1 mg/kg i.v.), butorphanol (0.015 mg/kg i.v.), ketamine (2.2 mg/kg i.v.) and guaiphenezine (25 mg/kg i.v.). The jenny was intubated using a 14 mm endotracheal tube and anesthesia was maintained with isoflurane (ET concentration 1.4 vol. % with oxygen as a carrying gas). On the left side of the neck, the hair was shaven and the skin prepared aseptically. After the skin incision, which was slightly proximal from the thoracic aperture, *vena jugularis sinistra* was prepared. The tip of a unipolar electrode was inserted under ultrasonographic control through the *vena jugularis* to the right ventricular apex. Although many attempts were made, a sufficient stimulation threshold was not found. During the procedure, periods of asystoly occurred, which were repeatedly resolved by indirect cardiac massage until the spontaneous heart rhythm was restored. The unipolar electrode was changed to bipolar with passive fixation (TIR 60 BP, Biotronik, Berlin, Germany). The length of the lead was 60 cm. The tip of the electrode was properly positioned and fastened in trabeculae of the ventricular myocardium. The proximal part of the lead was ligated to the surrounding tissue and connected to the pulse generator. A stimulation threshold was found at 0.7 V and 0.5 ms, with R waves of 7 mV. The pacemaker Kairos S (Biotronik, Berlin, Germany) was inserted into a subcutaneous pocket, which was created in the prescapular region. The subcutaneous tissue and skin were closed in a routine manner. The pacemaker was programmed to VVI mode with a minimal ventricular rate of 40 ppm (pulses per minute), a pulse amplitude of 4.8 V, a pulse width of 1.0 ms and sensing amplitude 2.5 mV. Ventricular stimulation 1:1 was confirmed by electrocardiography. Recovery from the general anaesthesia was uneventful.

The next day a mild swelling of the pacemaker surrounding tissue had developed. ECG confirmed regular heart rate and effective stimulation. Antibiotic and anti-inflammatory treatment was continued for three days (penicillin 30,000 IU/kg i.v. q8 h, gentamicin 6.6 mg/kg i.v. q24 h, flunixin 1.1 mg/kg i.v. q24 h). The jenny appeared alert and no syncopal episodes were observed. 

Six days after the surgery, an ECG check was performed. It was found that the pacemaker stimulation was without response and a complete AV block was present. The heart had intrinsic ventricular activity with a frequency of 53 bpm. Lead dislodgement was confirmed by measurement of sensing parameters.

Nine days after the first surgery, the jenny was subjected to a second procedure under general anaesthesia to correct the position of the electrode using the same anaesthetic protocol. The pacemaker pocket was reopened; the lead was disconnected and its reposition was guided by echocardiography. The stimulation threshold was found at 0.6 V at a pulse width of 0.5 ms and R waves of 11 mV. The electrode was connected back to the pulse generator and the pacemaker returned to the pocket. The pocket was then sutured in layers and the surgical wound was covered with a sterile dressing. The pacemaker was set to the same values as written above. Recovery from surgery was without any complications. The next day, ECG confirmed a full response to the pacemaker stimulation.

Mild swelling around the pacemaker pocket developed again and, therefore, antibiotic and anti-inflammatory medication was prolonged. Eight days after the second surgery, the jenny exhibited lethargy and fever. A complete blood count revealed leukocytosis (23 × 10^9^/L, reference range 5–14.5 × 10^9^/L). Infection of the pacemaker pocket was diagnosed. A mild exudation appeared from the wound and a sample was taken for microbial culture. *Staphylococcus aureus* resistant to penicillin and gentamicin was isolated, and the antibiotics were changed to cefquinome (2 mg/kg i.m. q12 h). Wound-cleaning was provided on a daily basis with povidone iodine solution. The wound secretion became purulent and the area around the wound was painful; surgical revision was indicated. The surgery (12 days after pacemaker reposition) was performed under analgo-sedation (diazepam 0.14 mg/kg i.v., butorphanol 0.01 mg/kg i.v., romifidine 0.02 mg/kg i.v.). After opening the pacemaker pocket, the pulse generator was disconnected and removed and the cavity was thoroughly flushed with povidone iodine solution. During this procedure, unintentional displacement of the electrode occurred and the whole pacemaker stimulation system had to be removed. The wound was closed with a one position stitch and left to heal by secondary intention.

Weakness and syncopal episodes occurred again. The jenny was weak and lethargic, with a heart rate around 50–60 bpm and irregular rhythm. The wound was cleaned and lavaged with povidon iodine solution daily. Cefquinom and phenylbutazone were administrated for another 8 days. Wound-healing was satisfactory; the heart rhythm continued to be irregular.

Sixteen days after removal of the stimulation system, the jenny was subjected to the implantation of a new pacemaker, which was performed on the contralateral site of the neck. Surgical procedure was performed in the same way as described above. The pacemaker pocket was flushed with antibiotic solution (neomycin, bacitracin), and then closed routinely. Pacemaker Kairos S (Biotronik, Berlin, Germany) was set to VVI mode, a basal rate of 40 ppm, a pulse amplitude of 4.8 V, a pulse width of 1.0 ms and sensing amplitude of 2.5 mV. The stimulation threshold was 0.7 V at 0.5 ms and with R waves of 10 mV.

Over the following days, the clinical status of the jenny was very good; only mild swelling at the site of the surgical wound was noticed. Therefore, antibiotic and nonsteroidal medication was continued for 2 weeks. After that, the pacemaker function was analysed. Considering the high energy requirement for ventricular pacing, the pacemaker was reprogrammed to 2.4 V, 0.5 ms and 2.5 mV. The jenny was discharged 3 weeks after successful reimplantation. 

### 3.3. Follow Up

The jenny was checked every six months to ensure the device was functional and performing appropriately. After three years, a regular check-up was done every 12 months. The jenny was used for animal-assisted therapy, prospered well and no side effects were observed.

Eight years later, during the regular check of the pacemaker, it was found that the pacemaker battery was low and a pacemaker replacement would be required. The jenny was admitted to the clinic. Its body condition was very good (BCS 7/9), with a heart rate around 40 bpm, and examination of the blood showed no abnormalities. On echocardiography, the lead tip was visible in the right ventricular apex, the lead surface was smooth and no irregularities or valvular lesions were detected on the tricuspid valve.

A surgical procedure was performed under general anaesthesia (premedication: penicillin 20,000 IU/kg i.v., gentamicin 6.6 mg/kg i.v., flunixin meglumine 1.1 mg/kg i.v., induction: xylazine 1.1 mg/kg i.v., ketamine 2.2 mg/kg i.v., diazepam 0.04 mg/kg i.v., anaesthesia maintained with isoflurane ET 1.4 vol. %). An incision was made in the skin above the pocket containing the pulse generator; the pacemaker pocket was opened and the device exposed. The lead was disconnected from the pulse generator. The old pacemaker was removed and replaced by the new one (Verity^TM^ ADx XL SC 5056, St. Jude Medical, Saint Paul, MN, USA). The cavity was closed in layers. Transient bradycardia had occurred during pacemaker disconnection (for approximately 2 min). The jenny recovered uneventfully. Medication after the procedure included flunixin meglumine (1.1 mg/kg i.v. q24 h) for 4 days and antibiotics for 7 days (penicillin 20,000 IU/kg i.v. q8 h, gentamicin 6.6 mg/kg i.v. q24 h). Because a phlebitis developed at the site of the intravenous catheter insertion, the catheter was removed and antibiotic treatment changed to enrofloxacin (5 mg/kg p.o. q24 h). The jenny was discharged after 3 weeks. Regular follow-up visits were done at one-year intervals.

**Figure 2:**
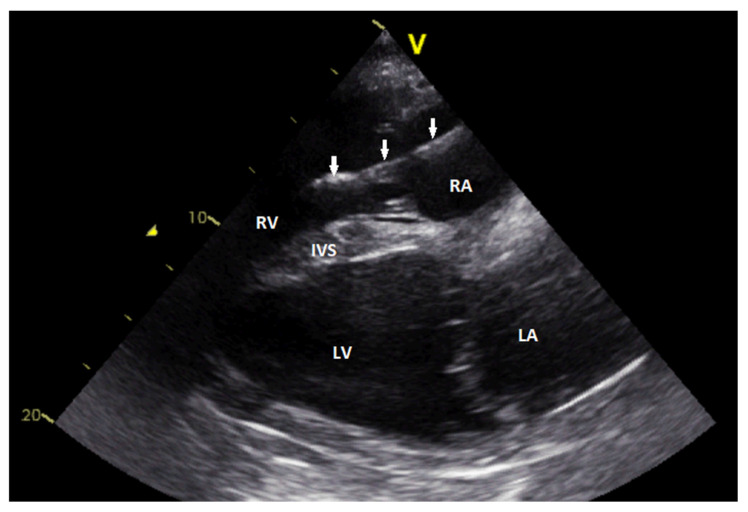
Echocardiographic image showing the right parasternal long-axis view. The lead (arrows) is visible in the right atrium and the right ventricle. The intraventricular part of the lead body shows slightly irregular surface (RA—rig ht atrium; RV—right ventricle; IVS—interventricular septum; LA—left atrium; LV—left ventricle).

During clinical examination before a routine vaccination 18 years after the initial implantation, an irregular heart rhythm was noticed. On that account, the jenny was admitted to the clinic. Physical examination revealed lethargic behavior, bradycardia and irregular heart rate; other organ systems were normal. Hematologic parameters were within reference ranges. ECG record confirmed a complete AV block with resting ventricular frequency 24 bpm and atrial rate around 50 bpm. Echocardiographic examination did not reveal any abnormalities (Figure 2), except mild tricuspid regurgitation visible in the color-flow doppler mode.

The pacemaker function was analysed and it was found that a pulse generator exchange was needed. The pulse generator was replaced under general anaesthesia, as described above. A firm fibrous capsule had developed around the device, so it had to be opened to expose the old pulse generator. It was replaced with pacemaker Etrinsa 8 DR-T (Biotronik, Berlin, Germany). A blind connector was inserted to the atrial channel of this pacemaker. The stimulation threshold was found at 0.9 V and 0.4 ms, with R waves of 8 mV. The pacemaker was programmed to VVI mode, basic rate 45 ppm, pulse amplitude 3.0 V, pulse width 0.4 ms and sensing amplitude 2.5 mV. Before closing the pacemaker pocket, the cavity was flushed with amikacin solution. Antibiotics (enrofloxacin 5 mg/kg p.o. q24 h) were administered for 5 days and ketoprofen (2 mg/kg i.v. q24 h) for 2 days.

**Figure 3:**
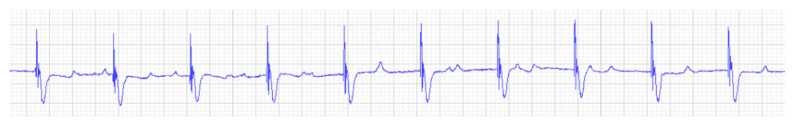
Base apex electrocardiogram with ventricular pacing shows regular rhythm with ventricular rate of 45 bpm. Ventricular pacing spikes precede each QRS complex (25 mm/s, 1 cm/mV).

Eight days after the procedure, a non-painful swelling around the pacemaker pocket developed. Ultrasonographic examination revealed a seroma formation at the site of the surgical wound. Non-steroidal anti-inflammatory gel was applied topically and enrofloxacin was given orally for 2 weeks. The stitches were removed on the ninth day after surgery. The wound was clean, without secretion, and the swelling gradually subsided. Before discharging the jenny, a cardiologic examination was performed (Figure 3). The pacemaker worked properly; the jenny was reliant on pacemaker stimulation from 86%. Battery lifespan was estimated to be 9 years.

## 4. Discussion

In horses, a complete AV block is uncommon and has been associated with inflammatory or degenerative diseases of the myocardial conducting tissues [10,15]. Pibarot et al. [5] reported a complete AV block in an 8-month-old female Jerusalem donkey, which started to have syncopal episodes at the age of 5 months. They assumed congenital aetiology in this particular case, because of the early appearance of the symptoms and the absence of clinical, haematological, biochemical and echocardiographic signs of chronic or acute cardiac disease. A familiar history of syncope was reported by Decloedt [16] in one donkey with several siblings suffering from episodes of collapse, suggesting a hereditary syndrome. Congenital aetiology could also be considered in our patient, when the onset of clinical signs started at the age of 4 months. However, partial improvement after phenylbutazone treatment may be indicative of inflammatory aetiology, although no clinical signs of any infectious disease were present. Serum troponin I measurement and other diagnostic tests were not performed due to strict financial constraints. 

Symptomatic bradydysrhythmias, such as third-degree AV block, are the major indications for permanent pacemaker implantation. A transvenous implantation technique in horses was described in detail by van Loon at al. [2]. Although the standing position is preferably used in horses [2,4], we decided to perform the surgery under general anaesthesia in lateral recumbency to ensure a better surgical approach and to avoid a collapse of the patient during the procedure. The jugular vein was used for lead insertion. Positioning of the lead was guided by echocardiography and the correct position was confirmed by measuring the electrical characteristics of the lead. 

Temporary transvenous pacing is used to avoid a syncope prior to the permanent implantation of a pacemaker [5,17]. Unfortunately, a temporary pacing system was not available in our case, and the lack of this equipment resulted in episodes of asystole during the first surgical intervention.

Although the transvenous implantation of a cardiac pacemaker is a relatively safe and simple procedure, there could be complications associated with it. Lead displacement is one of the most common major complications in both dogs and humans [7,11,13,18,19,20]. In our patient, lead dislodgement occurred in the early postoperative period and required correction of the lead position because of loss of capture. Thoracic radiography or fluoroscopy is used in small animals to verify a change in lead position [17]. Capture failure in the jenny was confirmed by surface ECG and the measurement of sensing parameters. The reintervention was performed nine days after the initial pacemaker implantation. 

Another major complication in our patient was pacemaker pocket infection. Swelling and secretion in the surgical wound occurred a few days after lead reposition. The jenny developed a fever and became lethargic. Despite prolonged antibiotic treatment, the pacemaker infection worsened. *Staphylococcus aureus* was isolated from the wound secretion. 

Cardiac implantable device infection is a serious problem even in human medicine [13,21]. Early reintervention is reported as a strong risk factor for later development of device infection [13]. Bacterial inoculation often occurs as a result of bacterial colonisation of the operative site at the time of pacemaker implantation. The *Staphylococcus* species from the skin may contaminate the wound, most likely during pocket formation, which later causes pocket infection [13]. The reported rate of infection in dogs varies from 1% to 5% [7,18,19,20,22], and *Staphylococcus* spp. is also the most commonly cultured isolate [20,22]. Treatment of the pacemaker pocket infection in our patient included complete system removal and prolonged antibiotic therapy. The new implantation was performed contralaterally, after complete wound-healing.

The jenny was used for animal-assisted therapy and was only ridden by children. There was no problem with this low-intensity exercise, although the VVI mode does not allow rate-adaptive pacing. A single-chamber pacemaker was sufficient for this type of mild exercise and prevented syncopes in our patient. The jenny was never used for breeding.

Follow-up visits to check the pacemaker function should be performed regularly. Pacemaker batteries are designed to have a predictable lifespan, which can be monitored by their cell voltage and cell impedance. The lifespan of a pulse generator is largely dependent on percent pacing, programmed voltage and pulse width and electrical pacing impedances. Average clinical longevity in human VVI pacemakers is reported to be around 7 years [23,24]. In our patient, the pacemaker checks were performed once a year. Surface ECG and pacing and sensing parameters of the pacemaker were measured. However, before the second pacemaker exchange, we missed the pulse generator end-of-life and the battery was completely discharged. Therefore, it would be advisable to shorten the intervals of the follow-up visits towards the end of the battery life.

The pulse generator exchange was performed twice at nine-year intervals. The procedure was associated with a localised reaction around the pacemaker device, which was resolved within a few days. Seroma formation is reported as a common minor complication in dogs and horses, and usually requires no treatment [2,7,18,19]. 

Long-term follow-up revealed no abnormalities on echocardiographic examination and successful pacing and sensing of the ventricle were obtained.

## 5. Conclusions 

This case report describes a successful transvenous pacemaker implantation in a jenny with a complete AV block. Major complications associated with the procedure were lead dislodgement and pacemaker pocket infection. However, the long-term outcome was satisfactory. The jenny showed improvement in heart function and quality of life after pacemaker implantation. 

Regular pacemaker checks should be undertaken to evaluate pacemaker function and battery condition. A more frequent follow up is necessary as the device approaches the end of its battery life. 

## Data Availability

Not applicable.
